# Editorial: Health in Afghanistan. Some insights from socio-epidemiological research

**DOI:** 10.3389/fpubh.2024.1367951

**Published:** 2024-02-27

**Authors:** Uwe H. Bittlingmayer, Stefanie Harsch, Diana Sahrai

**Affiliations:** University of Education Freiburg, Freiburg, Germany

**Keywords:** health in Afghanistan, health inequalities, socio-epidemiology in Afghanistan, COVID-19, healthcare in Afghanistan

## Introduction

“Health, Inequalities, and Diversity in Afghanistan.” was the initial title of this Research Topic, which was planned during the global pandemic in the spring of 2021. However, when the Taliban seized power in August 2021, the situation in Afghanistan changed fundamentally, affecting the health and wellbeing of its people as well as research. As a result, we were first hesitant to publish a call for abstracts on this Research Topic. However, despite the limited conditions for scientific work to meet international standards since August 2021, we decided to proceed with this Research Topic due to the encouragement of Afghan researchers, lecturers, and public health experts both inside and outside Afghanistan. We aimed to maintain collaboration and international research on health in Afghanistan. As all four peer-reviewed articles in the Research Topic are based on socio-epidemiological research and provide insights into various perspectives on diseases and attitudes of Afghan patients and the Afghan population, we have revised the title of this Research Topic to “*Health in Afghanistan. Some insights from socio-epidemiological research*” which more accurately describes the content.

## The contributions of the Research Topic

Although it is evident that the health of the Afghan population faces numerous challenges, there is nevertheless a sense of normalcy in everyday life where individuals cope with illness and disabilities. Afghanistan is also impacted by larger global trends, almost independent of socio-political circumstances. The scoping study by Neyazi et al. reflects that by analyzing the recent trends in non-communicable diseases (predominantly cardiovascular diseases, cancers, diabetes, and chronic obstructive diseases) in Afghanistan, which contribute to the burden of diseases in Afghanistan increased steadily. According to the World Health Organization's Regional Office for the Eastern Mediterranean, Afghanistan, Egypt, and Yemen have the highest burden of NCDs, with ~800 deaths per 100,000 people. Furthermore, individuals aged 30–70 years face a mortality risk of over 30% from cardiovascular disease, cancer, diabetes, or chronic respiratory disease (1). According to the report by the Commission on Non-communicable Diseases, Injuries and Poverty, established by the Ministry of Public Health of Afghanistan, stated that in 2016, 45.4% of deaths were caused by NCDs (2). Neyazi et al. provide a comprehensive scoping review of strategies to address non-communicable diseases in 15 countries. They identified 35 strategies with a strong focus on unhealthy diets and smoking. In a second step, the authors analyzed whether the NCD strategy of the former Afghan Ministry of Public Health adequately reflects the international state of the art, identified shortfalls, and provided recommendations for the most relevant causes of NCDs. However, it is noteworthy that the “causes of the causes,” like Michael Marmot mentioned, such as poverty, discrimination, and lack of access to primary healthcare, are hardly discussed in the context of public health strategies (3, 4).

The second article is located in the field of public health-analysis of communicable diseases. Saeed et al. analyze the Afghan population's knowledge, attitudes, and practices concerning COVID-19 in a cross-sectional survey across the 34 provinces conducted in spring 2021. There has been broad consent that COVID-19 has had a significant impact on the Afghan population, and Saeed et al. provide further insights on the Research Topic. On the one hand, the knowledge about COVID-19, as well as the attitudes and practices of the vast majority, reflect the recommendations of WHO and the Afghan authorities. On the other hand, the pandemic has affected the economic basis of many households. According to Saeed et al., 44% of the respondents have reported a reduced monthly salary, which mirrors the strong economic impact of the pandemic on the Afghan economy.

A third article addresses an important communicable disease in Afghanistan. Essar et al. analyze the knowledge, attitudes, and practices toward tuberculosis among hospital outpatients who visited the hospital for any health-related reasons and live in Kabul. With an estimated 13,000 tuberculosis-associated deaths annually, this disease is highly relevant for public health strategies. However, according to a cross-sectional survey conducted by Essar et al. between January and March 2022, respondents demonstrated surprisingly good knowledge, attitudes, and practices regarding tuberculosis. The study identified predictors of adequate awareness of the danger of tuberculosis, such as income, being young, and being male.

The fourth article discusses a public health issue of a more general nature. Shah et al. analyze the concerns of the Afghan population arising from the withdrawal of the NATO military forces. They present exploratory data from an online survey disseminated through social media and conducted on September 15th, 2021, immediately after the Taliban takeover. The questionnaire revealed that most Afghans expressed strong concerns about the withdrawal of the foreign troops, with females expressing significantly stronger concerns than males. Furthermore, most respondents expressed concerns about their mental health after the withdrawal.

## Public health in Afghanistan—final remarks

All four articles provide insights into the state of healthcare in Afghanistan at the end of the Islamic Republic of Afghanistan and during the transition to the Islamic Emirate of Afghanistan. Since the Taliban regained control, there have been multiple predictors of the full collapse of the Afghan healthcare system and the public health sector, such as the drastically increased poverty rate and the visible brain drain, particularly among medical doctors and medical staff members. Additionally, limitations have been imposed on women, pushing them out of the labor market. However, discussions about these issues in Afghanistan are often speculative and only refer to the urban population, a minority. In the current situation, there is hardly any doubt that health monitoring in Afghanistan is visibly limited, and the socio-economic situation remains poor (even though there has been some progress in 2023). The reduction in overall expenditure has significantly impacted health spending (5). Therefore, one of the most important tasks is to ensure that Afghans and the health of Afghanistan's population are not forgotten after the withdrawal of the NATO-led military forces and the shift of global media attention elsewhere.

## Author contributions

UB: Writing—original draft, Writing—review & editing. SH: Writing—original draft, Writing—review & editing. DS: Writing—original draft, Writing—review & editing.
